# Mouse Strain– and Charge-Dependent Vessel Permeability of Nanoparticles at the Lower Size Limit

**DOI:** 10.3389/fchem.2022.944556

**Published:** 2022-07-18

**Authors:** Haoran Chen, Yu-Cheng Liu, Zhiming Zhang, Moxin Li, Lidong Du, Pei-Chun Wu, Wai-How Chong, Fuzeng Ren, Weiming Zheng, Tzu-Ming Liu

**Affiliations:** ^1^ Institute of Translational Medicine, Faculty of Health Sciences, University of Macau, Taipa, Macau SAR, China; ^2^ Department of Materials Science and Engineering, Southern University of Science and Technology, Shenzhen, China; ^3^ Translational Medicine R&D Center, Zhuhai UM Science and Technology Research Institute, Zhuhai, China; ^4^ MOE Frontiers Science Center for Precision Oncology, University of Macau, Taipa, Macau SAR, China

**Keywords:** nanoparticles, drug delivery, lower size limit, vascular permeability, mouse strain–dependent, two-photon microscopy, charge-dependent

## Abstract

Remarkable advancement has been made in the application of nanoparticles (NPs) for cancer therapy. Although NPs have been favorably delivered into tumors by taking advantage of the enhanced permeation and retention (EPR) effect, several physiological barriers present within tumors tend to restrict the diffusion of NPs. To overcome this, one of the strategies is to design NPs that can reach lower size limits to improve tumor penetration without being rapidly cleared out by the body. Several attempts have been made to achieve this, such as selecting appropriate nanocarriers and modifying surface properties. While many studies focus on the optimal design of NPs, the influence of mouse strains on the effectiveness of NPs remains unknown. Therefore, this study aimed to assess whether the vascular permeability of NPs near the lower size limit differs among mouse strains. We found that the vessel permeability of dextran NPs was size-dependent and dextran NPs with a size below 15 nm exhibited leakage from postcapillary venules in all strains. Most importantly, the leakage rate of 8-nm fluorescein isothiocyanate dextran was significantly higher in the BALB/c mouse strain than in other strains. This strain dependence was not observed in slightly positive TRITC-dextran with comparable sizes. Our results indicate that the influence on mouse strains needs to be taken into account for the evaluation of NPs near the lower size limit.

## Introduction

Over the years, nanotechnology has been widely applied to cancer treatments such as chemotherapy and targeted therapy, referred to as cancer nanomedicine. Compared to conventional therapeutic molecules, cancer nanomedicines exhibit several advantages (Shi et al., 2010; Albanese et al., 2012; Shi et al., 2017). For example, they can reach tumor sites selectively, which subsequently increases efficacy and reduces systemic toxicity. By using nanocarriers, the pharmacological properties of therapeutic molecules can be improved, such as their solubility/biocompatibility and circulating half-life. The release of drugs can also be triggered by stimulus, enzymatic activity, or pH variation in a stable manner. Nowadays, a number of cancer nanomedicines have been approved for clinical use, with many more under clinical investigation.

It is well known that the accumulation of therapeutic nanoparticles (NPs) in solid tumors is through the enhanced permeation and retention (EPR) effect (Gerlowski and Jain, 1986; Matsumura and Maeda, 1986; Maeda et al., 2013; Wang et al., 2022). This is most likely due to leaky tumor vasculature and inefficient lymphatic drainage. However, although therapeutic NPs can extravasate from tumor blood vessels, their diffusion is likely to be restricted by the dense extracellular matrix (ECM) composed of collagen fibers and other proteins (Jain and Stylianopoulos, 2010). The elevated interstitial fluid pressure (IFP), which is caused by the lack of functional lymphatic vessels and the hyperpermeability of abnormal vasculature, also reduces the convective transport of NPs (Heldin et al., 2004). Furthermore, the vascular port size of metastatic brain tumors is found to be only ∼9 nm in diameter, which is 10-fold smaller than that in glioblastomas, leading to reduced vessel permeability of NPs (Mittapalli et al., 2017). To overcome those physiological barriers and enhance tumor penetration, one feasible strategy is to utilize NPs with a size of around 10 nm (Wong et al., 2011). While normalization of tumor vasculature with anti-angiogenic therapy was used to decrease the pore size of the vessel walls, 12 nm NPs also exhibited better diffusion in tumors in comparison with 125 nm NPs (Chauhan et al., 2012).

In general, NPs with a size larger than 20 nm are used to avoid renal clearance (Choi et al., 2011; Shilo et al., 2012) and interstitial/lymphatic fenestration (Moghimi et al., 2005). Actually, the lower size limit of NPs can be further decreased using properly designed nanocarriers without risking renal clearance. It has been reported that quantum dots with a hydrodynamic diameter of 8 nm did not exhibit renal excretion but liver accumulation at 4 hours post intravenous injection (Soo Choi et al., 2007). Albumin with an effective hydrodynamic diameter of 7.2 nm can also be a nanocarrier candidate which allows long circulation and enables extravasation into tumors but not into normal tissues (Kratz et al., 2011; Kianfar, 2021). In addition to size, other properties of NPs, including particle material, shape, surface charge, stiffness, and surface modification, could also affect their biodistribution, vessel permeability, cellular uptake, and clearance rate (Longmire et al., 2008; Petros and DeSimone, 2010; Albanese et al., 2012; Sun et al., 2014; Anchordoquy et al., 2017). For example, positively charged NPs with a size of 6–8 nm are more readily filtered out through the glomerular capillary than negatively charged nanoparticles with comparable sizes (Longmire et al., 2008). Positively charged NPs are more easily internalized than neutral and negatively charged NPs, while negatively charged NPs have a longer circulation half-life.

Numerous studies have focused on the optimal combination of particle material, size, surface charge, and other properties to improve the therapeutic potential of NPs against tumors. However, the effect of mouse strains on the characteristic evaluation of small-sized NPs has not been investigated yet. Therefore, in this study, we explored the influence of three mouse strains (BALB/c, C2J, and ICR) on the vessel permeability of NPs using two-photon microscopy. To visualize the blood stream *in vivo*, various sizes of dextran NPs ranging from 40 kDa (8.6 nm) to 2000 kDa (53 nm), which were labeled with fluorescein isothiocyanate (FITC) and tetramethylrhodamine (TRITC), respectively, were used as angiography tracers. We found that the vessel permeability of dextran NPs was size-dependent, and dextran NPs with a size below 15 nm exhibited leakage from postcapillary venules in all strains. Most importantly, the leakage of negatively charged FITC-dextran at 8 nm diameter was significantly higher in the BALB/c mouse strain than that in other strains. But the difference in vascular permeability among mouse strains was not observed in slightly positive TRITC-dextran with comparable sizes. Further study will be required to discover the mechanisms underlying the observed differences among mouse strains.

## Materials and Methods

### Mice

All animal experiments were conducted following protocols (UMARE0312016) approved by the Animal Ethics Committees, University of Macau. Six- to eight-week-old male BALB/c, C57BL/6-c2J (C2J), and ICR mice were bred in the Animal Facility at the Faculty of Health Sciences.

### Preparation of Fluorescent Dextran

Dextran with a molecular weight of 40, 70, 150, and 2000 kDa dyed with FITC or TRITC (Invitrogen) was used to prepare NPs with different sizes and surface charges. A total of 20 mg fluorescent-dextran powder was dissolved in 1 ml of phosphate-buffered saline (PBS) and sonicated in ice water for 30 min. The solution was sterile and filtered through 0.2-μm filters prior to use.

### Injection Setup

The injector containing a 1-ml syringe and a 27-G indwelling needle was connected with a PE tube. A total of 100 μl configured 2% wt fluorescence dextran was pre-loaded in the syringe, while anticoagulants were set in the needle.

To anesthetize the mice, isoflurane was used in our experiment due to its effectiveness, fewer side-effects, and rapid washout in continuous time-course imaging and prolonged experimental observations. Anesthesia was maintained with isoflurane vaporized at concentrations of 4% in the induction phase and at 0.8%–1.5% during prolonged experimental observations. The mice were under anesthesia until recovery, and their body temperature was maintained by a small warm bag. Mouse tail was treated with depilatory cream before the experiment for better observation effect. Their reflexes and vital signs were under continuous observation during the entire period (94–163 breaths/min, 325–780 beats/min, 37.5°C).

### Fluorescence Angiography

Skin on the ventral side of the ear was observed post fluorescent-dextran injection under a two-photon microscope. Images were obtained using a Nikon inverted multiphoton microscope (A1MP + Eclipse Ti-2E, Nikon Instrument Inc., Japan) with a water-immersed 40× and 1.15 NA objective. The 1,300-nm two-photon fluorescence imaging was used to locate the vessels. The excitation wavelengths for two-photon excitation of FITC and TRITC were set at 960 and 1,086 nm, respectively. The multiphoton time-course images at 323 × 323 μm field of view with 512 × 512 pixels were recorded for around 180 s. The frame rate was 0.52 fps. During recording, fluorescent dextran was injected using the prepared injector.

### Analytical Methodology of Vessel Permeability

**FIGURE 1:**
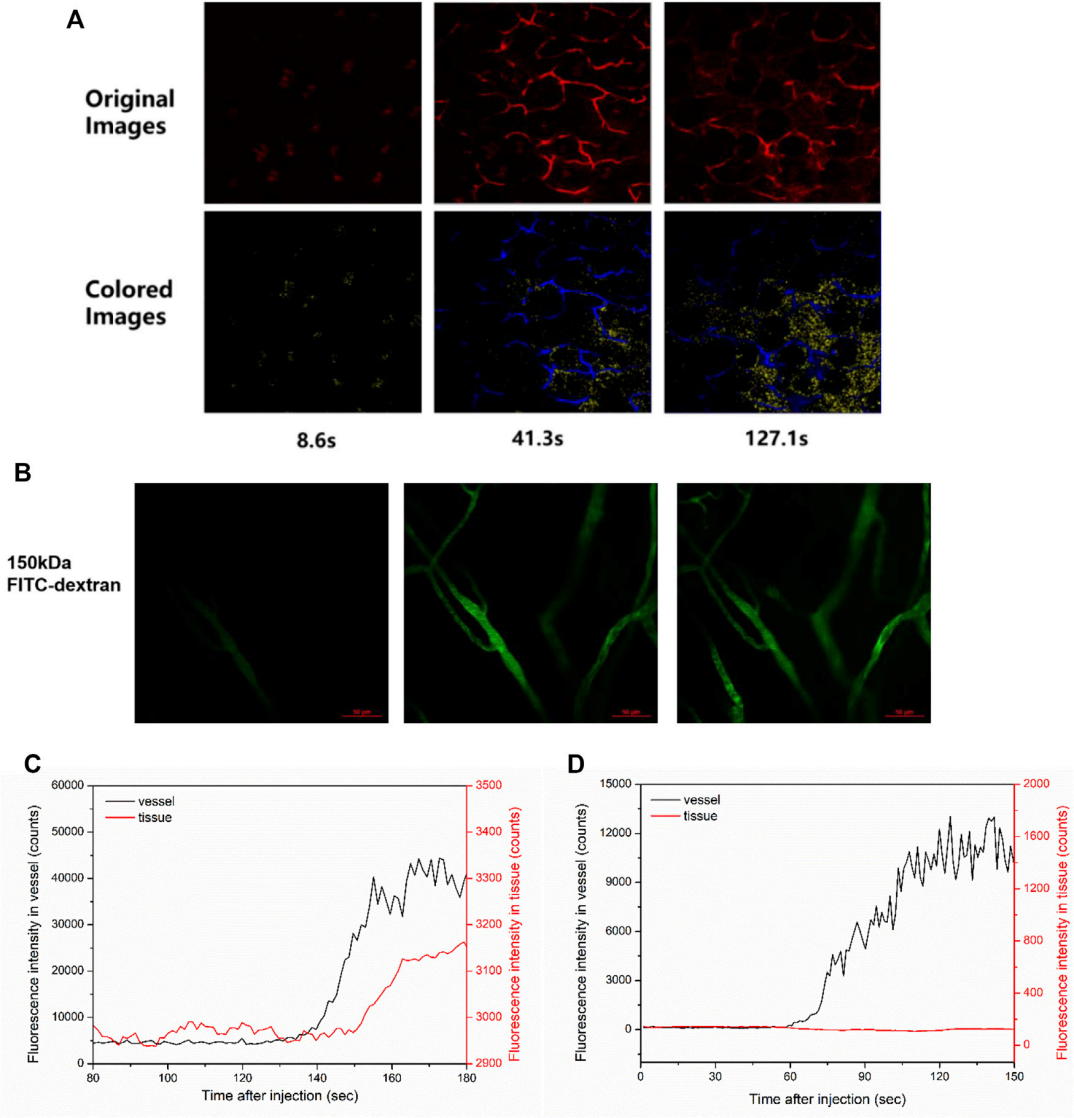
Vessel visualization and vascular permeability quantification. **(A)** Representative images of two-photon fluorescence angiography of 40 kDa TRITC-dextran tail-vein injected in ICR mice. The image acquisition time points were at 8.6, 41.3, and 127.1 s post injection, which showed the overall dextran perfusion in the vessel (blue color) and in the tissue (yellow color). **(B)** Representative images of two-photon fluorescence angiography of 150 kDa FITC-dextran injected in ICR mice. The series of images were taken at 15.5 s before flush, right after flush in, and 53.6 s after flush in. Scale bars: 50 μm. **(C,D)** The change of fluorescence intensity in a pair of the vessel point and the nearby tissue point measured **(C)** in the leaky region of **(A)** and **(D)** in the non-leaky region of **(B)**, respectively.

Software Image J was used to quantitatively analyze the permeability of mouse vessels. In each mouse, 30 pairs of the vessel measurement points and the adjacent tissue points were measured to obtain the fluorescence intensity over time. After fluorescent dextran was flushed, as in Figures 1A,B, the fluorescence intensity of vessels quickly reached a plateau and was constant for a while (Figure 1C). Meanwhile, the fluorescence intensity of tissue regions nearby gradually increased if the vessel was leaky (Figure 1D).

At the time point right after the vessel perfusion reached a plateau, the initial permeability rate was defined as the relative leaking ratio per second. The relative leaking ratio was calculated by frame-to-frame increase of tissue intensity ( $\Delta {\text{I}}_{\text{tissue}}$ ) divided by intravascular intensity ( ${\text{I}}_{\text{vessel}}$ ). Initial permeability rate can be formulated as:

$$\text{Initial permeability rate}=\;\frac{\Delta {\text{I}}_{\text{tissue}}}{\Delta \text{t}\times {\text{ I}}_{\text{vessel}}},$$ (1)

where △t is the frame interval.

For each mouse, 26–30 valid data pairs with dextran leaks were collected to calculate the average initial permeability rate.

### Statistical Analysis

Origin Pro software was used to perform statistical analysis of the data from conducted experiments. The descriptive statistics include the five-number summary (minimum, Q1, median, Q3, and maximum), interquartile range (IQR), outlier identification, sample mean, sample standard deviation, standard error, critical value, coefficient of variation, and 95% confidence interval. The significant difference hypothesis was tested by using either the one-way ANOVA with Tukey’s multiple comparison tests or the Mann–Whitney U test. Data were considered to be statistically significant when the *p*-value was less than 0.05.

## Results

### Vessel Visualization and Vascular Permeability Quantification

To visualize the perfusion of fluorescent nanomedicine in mice, we used two-photon microscopy for *in vivo* imaging with least invasiveness. Various molecular weights of dextran NPs (from 40 to 2000 kDa), labeled with fluorescent dye FITC and TRITC, respectively, were used as the contrast agents of angiography. The corresponding diameters are from 8.64 to 53.24 nm (see Table 1). The excitation wavelength was 960 and 1,086 nm for FITC-dextran and TRITC-dextran, respectively. The average laser power used for excitation was 19 mW, which showed no obvious tissue damage in the course of imaging.

**TABLE 1:** Molecular weight corresponding to diameter of nanoparticles.

Molecular weight	40 kDa (nm)	70 kDa (nm)	150 kDa (nm)	2,000 kDa (nm)
Diameter	8.64	10.17	15.87	53.24

Representative images of fluorescence in blood vessels post TRITC-dextran injection (Figure 1A) or FITC-dextran injection (Figure 1B) are shown. As for 2,000 kDa FITC-dextran, most of the tracers were retained in the blood post injection (Supplementary Figure S1). In contrast, obvious leakage could be observed for 40 kDa TRITC-dextran (Figure 1A). Notably, vascular leakage tends to occur at the postcapillary venules but not from capillaries (Nagy et al., 2008; Egawa et al., 2013). To measure the fluorescence intensity over time, 30 pairs of the vessel points and the adjacent tissue points were randomly obtained in each mouse and analyzed using Image J. For example, after 40 kDa TRITC-dextran was flushed in the ear vessels of ICR mice (Figure 1A), the fluorescence intensity of a vessel point quickly increased and reached a plateau (Figure 1C, black line). The change in fluorescence intensity of a tissue point nearby (red line) was used to define whether the vessel was leaky. Around the leaky vessels, the intensity of nearby tissue measurement points gradually elevated after injection (Figure 1C, red line). On the contrary, around the non-leaky vessels in ICR mice receiving 150 kDa FITC-dextran (Figure 1B), this intensity change was not observed (Figure 1D). In this study, we focused mainly on the leaky vessels to record the change in fluorescence intensity for the initial permeability rate. The calculation for the initial permeability rate was described in Eq. 1. The permeability rate is the increased fluorescence intensity in tissue areas divided by that in vessels. In our study, the average intensity in vessels is typically around 2,000 counts. Considering the minimum increase of one count, the minimal resolvable permeability rate is about 0.0005, which is therefore set as the threshold of the initial permeability rate. During data processing, we treated a measurement point as valid data only when its permeability rate was above this threshold.

To quantify the initial permeability rate of each mouse, we collected 30 valid data points to generate the box chart. Median, Q1, Q3, and IQR were used to calculate to the lower and upper bounds for a group of valid data. The lower and upper bounds for a group of samples were set at their median ± 1.5 × IQR. Any data that were less than the lower bound or more than the upper bound were considered as outliers and removed. Based on this, the number of qualified data obtained ranged from 25 to 30. Then the mean of initial permeability rate, standard deviation, and coefficient of variation were obtained for each BALB/c mouse injected with 40 kDa FITC-dextran (see Table 2). Given that there were 25–30 valid data points, the permeability data were considered to have a normal distribution. The lower and upper 95% confidence intervals could also be calculated. The value of the coefficient of variation was similar among most of the mice, excluding one mouse, which was most likely due to an individual difference. It indicated that the methods of data processing and analysis for the initial permeability rate in each mouse were accurate, precise, and reliable in this study.

**TABLE 2:** Statistical analysis of initial permeability rate of each BALB/c mouse injected with 40 kDa FITC-dextran.

Mouse ID	Valid data number	Mean	Standard error of mean	Coefficient of variation	Lower 95% confidence interval of mean	Upper 95% confidence interval of mean
1	30	0.00464	0.00041	0.48352	0.00381	0.00548
2	28	0.00211	0.00028	0.70465	0.00153	0.00269
3	29	0.00352	0.00025	0.38281	0.00301	0.00403
4	30	0.00376	0.00031	0.44559	0.00313	0.00439
5	30	0.00323	0.00031	0.52544	0.00259	0.00386

### Size Effects on Vascular Permeability of Fluorescent Dextran

**FIGURE 2:**
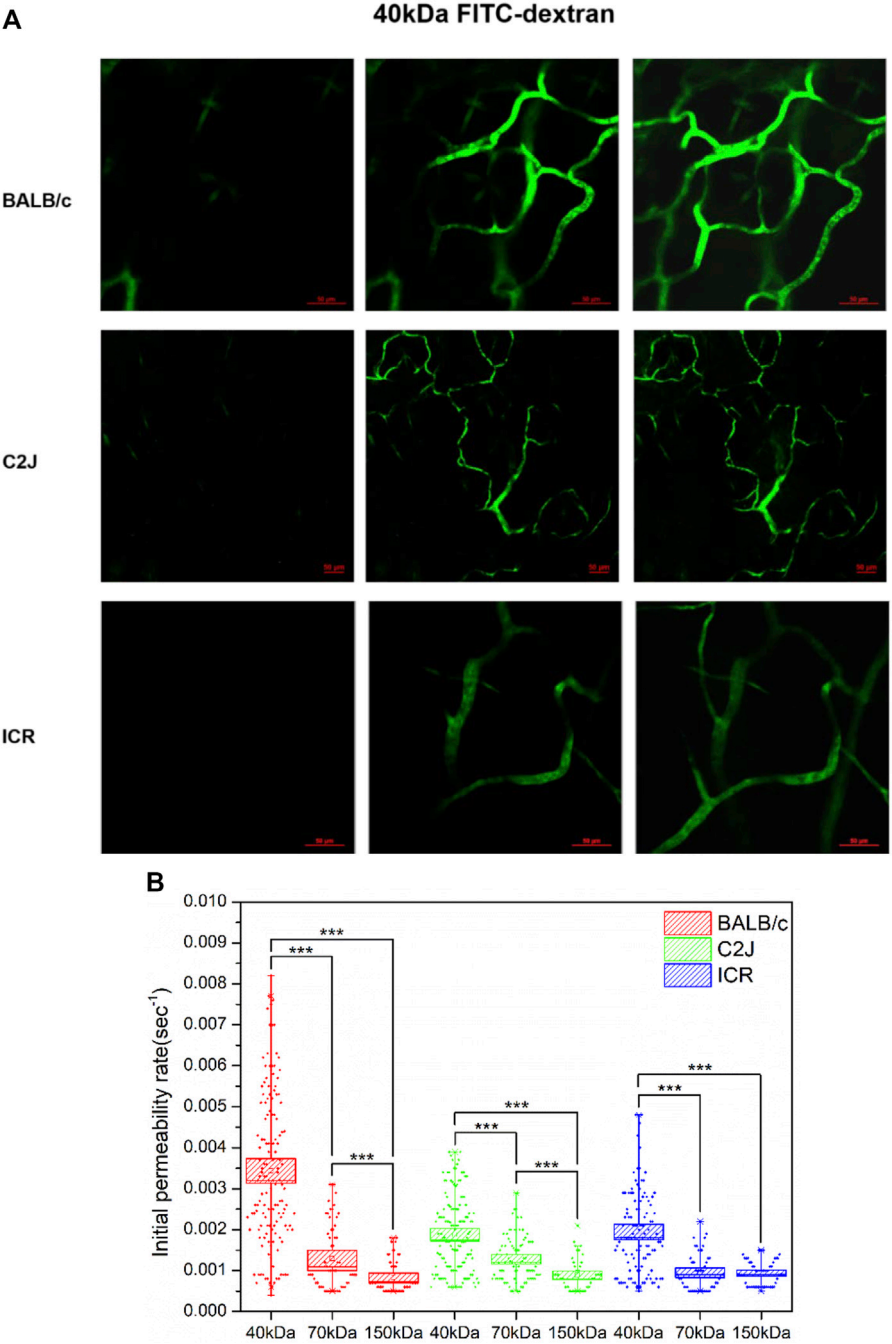
**(A)** Representative images of two-photon fluorescence angiography of 40 kDa FITC-dextran injected in three strains of mice, respectively. The series of images were taken at 15.4–20.7 s before injection (left column), right after dextran flush in (middle column) and at 57.2–86.6 s post injection (right column). Scale bars: 50 μm. **(B)** The initial permeability rate of FITC-dextran at 40, 70, and 150 kDa in three mouse strains of BALB/c, C2J, and ICR, respectively. The valid measurement data of each mouse within the same group were merged together to generate the box chart. The data are presented as mean ± SEM. Significant differences were analyzed using the one-way ANOVA with Tukey’s multiple comparison tests. ***p < 0.005.

Following the analysis aforementioned, the valid data of each mouse in the same group were plotted together to analyze the effect of size on vascular permeability of NPs. The vascular permeability of fluorescent dextran with sizes of 40 kDa (8.6 nm), 70 kDa (10.17 nm), and 150 kDa (15.87 nm) among three mouse strains was evaluated. Figure 2A demonstrates the representative images of 40 kDa FITC-dextran angiographs. In the BALB/c mouse model, all 40–150 kDa FITC-dextran leaked from postcapillary venules into normal tissues (Figure 2B). The initial permeability rate increased following the decrease in the size of FITC-dextran. Specifically, 40 kDa FITC-dextran (0.00344 ± 0.00015) exhibited significantly stronger leakage compared to 70 kDa FITC-dextran (0.00128 ± 0.0006) (*p* < 0.005). The initial permeability rate of 150 kDa FITC-dextran was the lowest, which was 0.00083 ± 0.00005. Size-dependent leakage can also be validated using the one-way ANOVA test (*p* < 0.005). A similar trend of leakage was also observed in the other two mouse strains.

### Strain Effects on Vascular Permeability of Fluorescent Dextran

**FIGURE 3:**
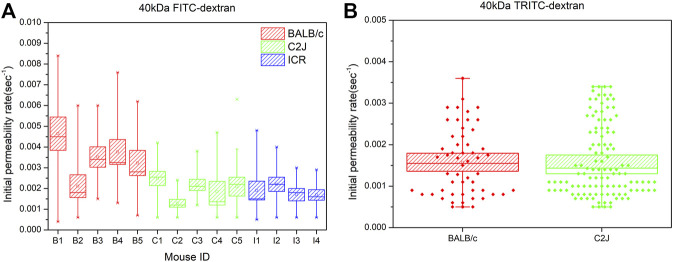
**(A)** Comparison of the initial permeability rates of 40 kDa FITC-dextran between BALB/c (B1-B5), C2J (C1-C5), and ICR (I1-I4) mouse models. Significant differences were analyzed using the Mann–Whitney U test. Data are presented as mean ± SEM **(B)** Comparison of the initial permeability rates of 40 kDa TRITC-dextran between BALB/c and C2J mouse models. The valid measurement data of each mouse within the same group were merged together to generate the box chart. The data are presented as mean ± SEM. Significant differences were analyzed using the one-way ANOVA with Tukey’s multiple comparison tests.

Then, we further analyzed whether mouse strains affected the vascular permeability of fluorescent dextran at various sizes. When mice were injected with 40 kDa FITC-dextran, the mean initial permeability rate in the BALB/c group tended to be higher than that in the C2J and ICR groups (Figure 3A). The significant difference hypothesis between BALB/c and the C2J mouse strain was analyzed using the Mann–Whitney U test (See Table 3). The alternative hypothesis was that the sample mean of BALB/c is higher than that of C2J. The calculated U value is 2, which is smaller than the critical value of 4 based on the 0.05 significant level in the one-tail test (one-tailed *p*-value = 0.0159). Therefore, we concluded that the BALB/c strain has a higher leakage of 40 kDa FITC-dextran than the C2J mouse strain. We also merged all the valid measurement data of each mouse within the same group together to compare the difference in the initial permeability rate among three groups of strains (Supplementary Figure S2A). Significantly higher leakage was also found in BALB/c mice in comparison with C2J (*p* < 0.005) and ICR (*p* < 0.005) by using the one-way ANOVA test with Tukey’s post-hoc analysis. These results indicated that the vascular permeability of 40 kDa FITC-dextran was strain-dependent and the leakages would happen more easily in the BALB/c mouse strain. In terms of 70 kDa FITC-dextran, however, statistical significance was only observed between BALB/c and ICR mice (0.00128 ± 0.00006 versus 0.00095 ± 0.00006, *p* < 0.05) (Supplementary Figure S2B). In addition, there was no notable difference in the initial permeability rate after administering 150 kDa FITC-dextran among the three mouse strains (Supplementary Figure S2C). We are aware that the number of mice was limited in the present study. Sufficient mouse numbers should be achieved to make the findings more convincing in future studies.

**TABLE 3:** Statistical analysis of difference in initial permeability rates between BALB/c and C2J mice post 40 kDa FITC-dextran injection by Mann–Whitney *U* test.

Group	BALB/c vs. C2J
Number	5
Sum rank of BALB/c	38
Sum rank of C2J	17
U value	2
Critical value of U at *p* < 0.1 (two tailed)	4
One- or two-tailed hypothesis	One-tailed
*p* value	0.0159

This comparison was also performed in the panel of slightly positive TRITC-dextran. 40 kDa TRITC-dextran in the BALB/c model had a initial permeability rate (0.00158 ± 0.00011) similar to that in the C2J model (0.00159 ± 0.000081) (Figure 3B). The difference in vascular permeability between BALB/c and C2J mice was not seen in TRITC-dextran with comparable sizes (*p* = 0.8967).

### Surface Charge Effects on Vascular Permeability of Fluorescent Dextran

**FIGURE 4:**
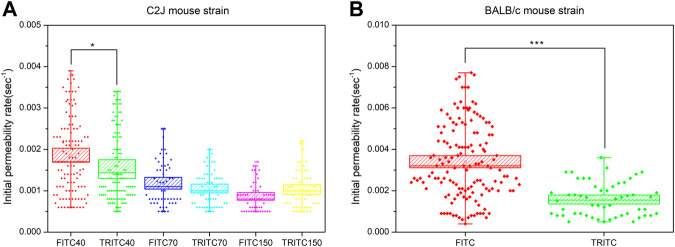
**(A)** Comparison of the initial permeability rates between FITC-dextran and TRITC-dextran at 40, 70, and 150 kDa in the C2J mouse strain **(B)** Comparison of initial permeability rates between 40 kDa FITC-dextran and 40 kDa TRITC-dextran in the BALB/c mouse strain. The valid measurement data of each mouse within the same group were merged together to generate the box chart. The data are presented as mean ± SEM. Significant differences were analyzed using the one-way ANOVA with Tukey’s multiple comparison tests. *p < 0.05, ***p < 0.005.

To assess the effects of the surface charge of NPs on vascular permeability, FITC-dextran and TRITC-dextran at various sizes were compared in the C2J mouse strain. A zeta potential measurement showed that FITC-dextran was negatively charged, while TRITC-dextran was neutral (see Supplementary Table S1). Here, we can see that negatively charged FITC-dextran at 40 kDa (0.00186 ± 0.00009) had a significantly higher initial permeability rate than neutral TRITC-dextran with comparable sizes (0.00159 ± 0.00008) (*p* < 0.05) (Figure 4A). In the 70 kDa panel, however, there is no significant difference in the initial permeability rate between FITC-dextran (0.00118 ± 0.00007) and TRITC-dextran (0.00106 ± 0.00005) (*p* = 0.1599). A similar pattern was found in 150 kDa dextran. In terms of the BALB/c mouse strain, a higher initial permeability rate was seen in negatively charged FITC-dextran (0.00340 ± 0.00015) than that of neutral TRITC-dextran at 40 kDa (0.00158 ± 0.00011) (*p* < 0.005) (Figure 4B). It suggests that surface charge of NPs is also one of the factors that affect the initial permeability rate of NPs with a size of 8.64 nm, which is consistent with other studies (Jallouli et al., 2007; Setyawati et al., 2013; Setyawati et al., 2015). Given that FITC-dextran but not TRITC-dextran showed significant leakage among mouse strains, we hypothesized that surface charge may be the factor that affects the initial permeability rate.

## Discussion

In this study, we used two-photon microscopy to observe and quantify the leakage of nanoparticles from the skin micro-vessels of mice. Vascular permeability was used as the evaluation index to compare the impact of mouse strains on nanoparticles at the lower size limit. Here, we demonstrated that the degree of vascular permeability into tissue is highly correlated with the size of nanoparticles. FITC-dextran with 40 kDa (8.6 nm) had significantly higher leakage than those of 70 kDa (10.17 nm) and 150 kDa (15.87 nm). A slight leakage was also found in FITC-dextran with 70 and 150 kDa, which was in contrast to a previous study revealing no leakage of FITC-dextran with a size above 70 kDa (Sarin, 2010; Egawa et al., 2013). This difference is probably because the method of initial permeability has a higher sensitivity to vessel leakage. We focused mainly on the leaky vessels instead of the whole tissue volumes. Despite this, the majority of dextran with a size above 70 kDa was retained in the blood in both our study and previous studies. It supports the result that there is a permeation restriction for NPs larger than 70 kDa as most plasma components such as albumin and transthyretin-tetramer are around 50–70 kDa.

The major finding of this study is the significantly higher leakage of negatively charged 40 kDa (8.6 nm) FITC-dextran in the BALB/c mouse compared with other mouse strains. At the vessel surface, the transport of NPs across the endothelium includes two major routes: 1) paracellular transport by diffusion through cell junctions and 2) transcellular transport mostly via caveolae-mediated vesicular transport (Cao et al., 2017; Sheth et al., 2021). Several molecules, including vascular endothelial growth factor (VEGF), thrombin, and histamine, can weaken junctional adhesions and cause the formation of cell junction gaps (Komarova and Malik, 2010). This change, therefore, increases the endothelial permeability of small NPs through paracellular transport. The lack of caveolin-1 (Cav-1), a structural protein of caveolae, also leads to increased permeability to plasma albumin in Cav-1-null mice (Razani et al., 2001). As the endothelial leakage of dextran was observed in a short period after injection, it was most likely achieved *via* paracellular transport but not transcellular transport. We therefore hypothesized that the increased vascular permeability of NPs was probably due to the varied expression levels of VEGF, thrombin, and histamine among three mouse strains. For example, the expression level of VEGF receptor 1 in BALB/c mice was found to be higher than that in C57BL/6 mice (Imoukhuede and Popel, 2012). The underlying mechanisms of mouse strain–dependent vessel permeability will be investigated in a follow-up study. Moreover, as we examined only the vascular permeability, it is one of the limitations of this study. It will be of interest to assess the difference in circulation half-life, tumor penetration, renal clearance, or tissue accumulation of NPs at a lower size limit among mouse strains, which may provide more information.

Notably, the strain-dependent leakage was not found in slightly positive TRITC NPs. It suggests that the change of surface charge may counteract the effect of mouse strain on the vascular permeability of NPs with a size of 8.64 nm. It is possible because the transport of positively charged NPs across the endothelium was less efficient, resulting in similar vascular permeability among three mouse strains. It has been reported that neutral glucose NPs remained mostly at the surface of endothelial cells, while their positively charged NP counterparts were able to cross the vascular barrier *via* the transcellular and paracellular routes (Jallouli et al., 2007; Zhang et al., 2020). Negatively charged Au NPs (24 nm) were found to cause higher endothelial leakage than their positively charged counterparts by the binding of VE-cadherin at the endothelial cell junction (Wang et al., 2018). Given the negatively charged glycocalyx on the surface of endothelial cells, negatively charged nanoparticles may have “bounced” along the cell membrane until they reached the cell junction. In contrast, positively charged nanoparticles are electrostatically attracted by the negatively charged glycocalyx and remain on the cell membrane, extending the residence time for subsequent endocytosis. Consistent with this, our study demonstrated that negatively charged FITC-dextran showed significantly higher leakage than slightly positive TRITC-dextran in either the BALB/c or C2J mouse models.

In conclusion, fluorescent dextran in a size lower than 150 kDa would leak from postcapillary venules post injection and this leakage was size-dependent. The increase in the initial permeability rate of 40 kDa FITC-dextran was found in the BALB/c mouse strain but not in other mouse strains. Therefore, the selection of mouse strain needs to be considered as well during the evaluation of anionic NPs at the lower size limit. This enhanced permeability rate was likely to be restrained by the change from negatively charged FITC to neutral TRITC.

## Data Availability

The raw data supporting the conclusion of this article will be made available by the authors, without undue reservation.
