# Supplementary material for: Mouse Strain– and Charge-Dependent Vessel Permeability of Nanoparticles at the Lower Size Limit
Source: Front Chem. 2022 Jul 18;10:944556. doi: 10.3389/fchem.2022.944556 (PMC9339680; doi:10.3389/fchem.2022.944556)
Supplement: Supplementary file 1 [file DataSheet1.docx]

Supplementary Material

**
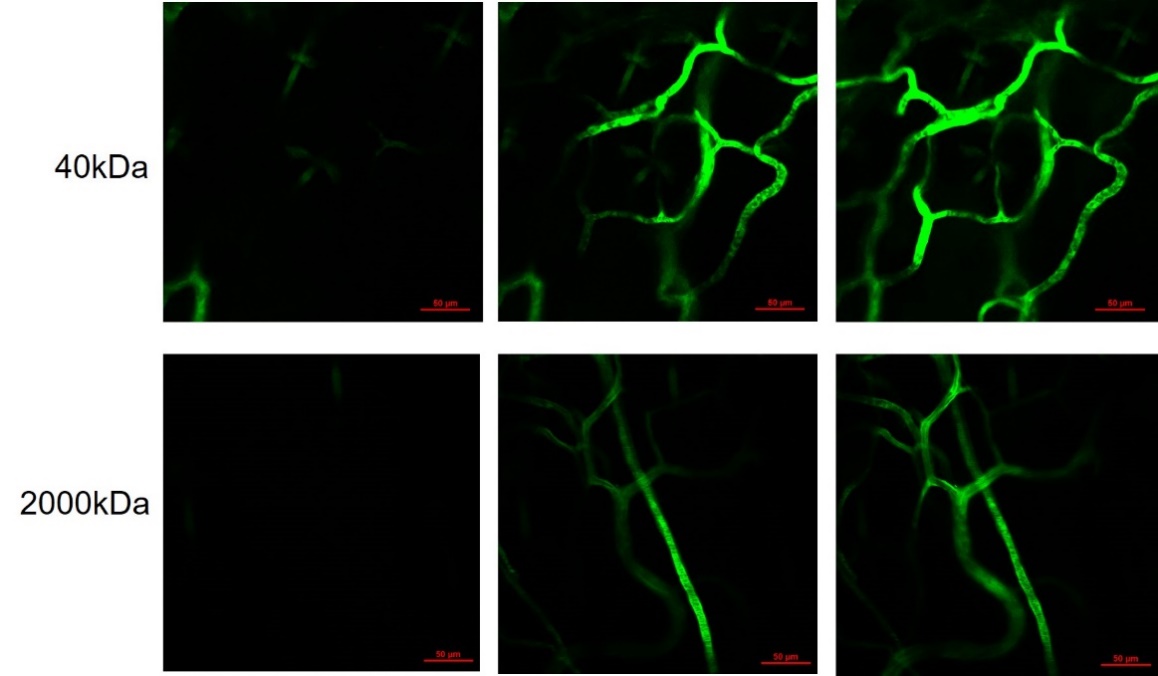
**

**Supplementary Figure 1** Representative images of two-photon fluorescence angiography of 2000 kDa FITC-dextran injected in BALB/c mice. The series of images were taken 26.8s before flush, right after flush in, and 55.5s after flush in time point**.**

A. B.







C.





**Supplementary Figure 2.** Comparison in initial permeability rate of FITC-dextran with (A) 40 kDa (Balb/C n=5, C2J n=5, ICR n=4), (B) 70 kDa (n=2) and (C) 150 kDa (n=2) among three mouse strains. The valid measurement data of each mouse within the same group was merged together to generate the box chart. Data are presented as mean ± SEM. Significant differences were calculated using the one-way ANOVA with Tukey’s multiple comparison tests. * P < 0.05, *** P < 0.005.

**Supplementary Table 1.** Mean zeta potential of FITC-dextran and TRITC-dextran

| Sample Name | T | ZP | Mob | Cond |
| --- | --- | --- | --- | --- |
|  | °C | mV | µmcm/Vs | mS/cm |
| 40kDa FITC-dextran | 25 | -0.717 | -0.05632 | 0.196 |
| 70kDa FITC-dextran | 25 | -7.91 | -0.6198 | 0.176 |
| 150kDa FITC-dextran | 25 | -15.1 | -1.182 | 0.117 |
| 40kDa TRITC-dextran | 25 | 0.112 | 0.008787 | 0.0205 |
